# Efficacy of traditional Chinese medicine combined with Western drugs for the treatment of non-proliferative diabetic retinopathy: a meta-analysis

**DOI:** 10.3389/fmed.2026.1767892

**Published:** 2026-04-22

**Authors:** Yinan Shao, Haotian Li, Simin Xu, Fan Gan, Xuemei Li

**Affiliations:** 1 Department of Ophthalmology, The First Affiliated Hospital of Baotou Medical College, Baotou, Inner Mongolia, China; 2 Department of General Surgery, The First Affiliated Hospital of Baotou Medical College, Baotou, Inner Mongolia, China; 3 Department of Ophthalmology, School of Optometry, Jiangxi Medical College, Nanchang University, Nanchang, Jiangxi, China

**Keywords:** Chinese medicines, diabetic retinopathy, general surgery, non-proliferative diabetic retinopathy, ophthalmology

## Abstract

**Objective:**

This study aimed to evaluate the efficacy of combination therapy with Chinese and Western medicine for non-proliferative diabetic retinopathy (NPDR).

**Methods:**

This systematic review was registered in the International Prospective Register of Systematic Reviews (PROSPERO) under registration number CRD420261357240. PubMed, China Knowledge Network, China Wanfang, and Embase databases were searched for literature on the combination of Chinese and Western medicines in the treatment of NPDR from the inception of each database. Two evaluators independently screened the literature, extracted data, and evaluated the risk of bias of the included studies, and the data were analyzed using the RevMan 5.3 software for statistical analysis.

**Results:**

Six relevant studies (total *n* = 1,127) were included after rigorous screening. Risk of bias assessment revealed that all included studies had high or unclear risk in at least two domains. GRADE assessment indicated low to moderate quality of evidence across outcomes. Compared with Western medicine monotherapy, combination therapy showed superior effects on visual acuity (mean difference (MD) = 0.09, 95% confidence interval (CI): 0.07–0.11, *I*^2^ = 0%, five studies), whole blood high-cut viscosity (MD = −0.55, 95% CI: −0.83 to −0.27, *I*^2^ = 88%, four studies), whole blood low-cut viscosity (MD = −0.61, 95% CI: −1.12 to −0.11, *I*^2^ = 89%, four studies), Traditional Chinese Medicine (TCM) symptom score (MD = −2.29, 95% CI: −4.26 to −0.32, *I*^2^ = 85%, three studies), and TCM symptom efficacy (odds ratio (OR) = 4.07, 95% CI: 1.37–12.10, *I*^2^ = 71%, three studies). Publication bias was not detected (Egger’s test *p* = 0.18). Clinical efficacy (OR = 2.96, 95% CI: 2.08–4.21, *I*^2^ = 39%, six studies) was consistently favorable across studies.

**Conclusion:**

The combination of Chinese and Western medicines in the treatment of NPDR can improve visual acuity, improve blood rheology, reduce the clinical symptoms of Traditional Chinese Medicine syndromes, increase the efficacy of TCM treatments, delay disease progression, alleviate the symptoms, and optimize patients’ quality of life, demonstrating remarkable effects and promising clinical application.

## Introduction

Diabetic retinopathy (DR) is one of the most common clinical complications of diabetes mellitus. DR affects 22.7% of the 537 million people with diabetes worldwide and has become an important cause of vision loss, causing inconvenience in patients’ lives and aggravating mental stress. Currently, there are approximately 140 million diabetic patients in China, and the number of DR patients is estimated to be 53 million, making the research of clinical drugs for treating DR a significant challenge for China.

Traditional Chinese Medicine (TCM) is a medical therapy that has been used in China for thousands of years. Studies have long confirmed that TCM can lower blood glucose, blood lipids, and other metabolic indices and has significant efficacy in managing diabetes and its complications (1). Some studies have suggested that TCM has a better intervention effect on DR (2, 3). In China’s clinical practice, not only is Chinese medicine commonly used in the treatment of DR, but Western medicine often incorporates Chinese medicine as a complementary therapy for managing DR. Currently, Western medicine uses anti-VEGF drugs for the treatment of DR, but the short half-life of these drugs requires frequent injections to maintain efficacy, the drugs are expensive, and the complications of vitreous cavity injections may increase in incidence due to the frequency of the injections.

DR can be categorized into two types: non-proliferative diabetic retinopathy (NPDR) and proliferative diabetic retinopathy (PDR). Patients with NPDR present early clinical signs and symptoms, which can be improved by active treatment; however, if treatment is not timely, NPDR may progress to proliferative DR, leading to severe visual impairment. The main method of treating DR with TCM in China is to use Chinese herbs to improve retinal microcirculation in NPDR and alleviate related symptoms. However, the effectiveness of TCM in the treatment of NPDR remains controversial. To clarify whether TCM can improve the clinical symptoms of NPDR patients and to explore the effectiveness of combining TCM with Western medicine in treating NPDR, the present analysis was conducted to provide more reference for the clinical treatment of NPDR.

## Methods

### Subject of the study

This systematic review and meta-analysis was conducted in accordance with the Preferred Reporting Items for Systematic Reviews and Meta-Analyses (PRISMA) 2020 statement. The protocol was prospectively registered in the International Prospective Register of Systematic Reviews (PROSPERO) under registration number CRD420261357240. Patients with non-proliferative diabetic retinopathy were identified based on clinical diagnostic criteria, with no restrictions on disease duration.

### Types of studies

Randomized controlled trials (RCTs) were included, in which patients with DR receiving a combination of Chinese and Western medicines were randomized into groups.

### Interventions

The experimental group received TCM in addition to conventional Western medicine, while the control group received conventional Western medicine alone.

### Inclusion criteria

Patients with NPDR who meet the relevant diagnostic criteria and are treated with a combination of Chinese and Western medicines were included. The Chinese medicine interventions could consist of herbal medicines, compound preparations, proprietary Chinese medicines, injections, or ionic introduction. Only randomized controlled trials were considered, and the control group consisted of patients with NPDR treated with Western medicine alone.

### Exclusion criteria

Studies were excluded if they were non-randomized controlled trials, did not meet the diagnostic criteria for NPDR, or had unclear diagnosis; involved invasive treatments such as surgery; included patients with other types of retinopathy; did not involve treatment with TCM; were duplicate publications; or were reviews, animal studies, expert opinions, or case reports. Additionally, studies were excluded if they had unreasonable methodological designs, inappropriate statistical methods, or contained data with serious errors.

### Indicators of outcome

The efficacy assessment criteria for visual acuity and fundus hemodynamics were based on established international and domestic standards, namely the “Severity Grading Criteria for Diabetic Retinopathy and Diabetic Macular Edema Disease” (4) and the current “Clinical Staging Criteria for Diabetic Retinopathy” in China. The criteria for evaluating the efficacy of TCM symptoms followed the “Guidelines for Clinical Research on New Chinese Medicines for Diabetic Retinopathy” (5), which was developed by the Ministry of Health of the People’s Republic of China in 2002.

### Literature search strategies

PubMed, China Knowledge Network, China Wanfang, and Embase databases were searched from database inception to November 2023. The search strategy was developed with assistance from a trained medical librarian. Chinese search terms included (“Traditional Chinese Medicine” OR “Chinese Herbal Medicine” OR “TCM”) AND (“Diabetic Retinopathy” OR “Non-proliferative Diabetic Retinopathy” OR “NPDR”) AND (“Randomized Controlled Trial” OR “Clinical Trial” OR “Efficacy”). English search terms included (“traditional Chinese medicine” OR “Chinese herbal medicine” OR “TCM”) AND (“diabetic retinopathy” OR “non-proliferative diabetic retinopathy” OR “NPDR”) AND (“randomized controlled trial” OR “RCT” OR “clinical trial”). An example PubMed search string was: ((“Medicine, Chinese Traditional”[Mesh] OR “Drugs, Chinese Herbal”[Mesh]) AND (“Diabetic Retinopathy”[Mesh] OR “non-proliferative diabetic retinopathy”[Title/Abstract])) AND (“Randomized Controlled Trial”[Publication Type] OR “controlled clinical trial”[Title/Abstract]). Searches were limited to human studies and articles published in English or Chinese.

### Quality assessment

The first screening of titles and abstracts was carried out simultaneously and independently by two researchers to remove non-compliant literature based on the inclusion and exclusion criteria. Following this, the second screening was performed by reading the full texts of literature that initially met the criteria, and the authors of eligible studies were contacted for quality evaluation. Third-party personnel were consulted for discussion of controversial literature. Finally, the literature data were extracted, including the title, authors, year, trial details, results, and other relevant information. The Cochrane risk of bias tool was used to assess the included studies, including randomization, allocation concealment, whether blinding was used, completeness of outcome indicators, whether the results of the study were selectively reported, and other biases. The results were categorized into three risk levels: “high risk,” “low risk,” and “uncertain.”

### Statistical analysis

Meta-analysis was performed using RevMan 5.4 and Stata 17.0. The effect model was selected according to the magnitude of heterogeneity, with heterogeneity *I*^2^ < 50% considered low, and a fixed-effects model (Mantel–Haenszel method) was used; *I*^2^ ≥ 50% indicated substantial heterogeneity, and a random-effects model (DerSimonian–Laird method) was used. For outcomes with I^2^ ≥ 50%, we performed subgroup analyses (stratified by treatment duration: ≤3 months vs. >3 months) and sensitivity analyses (leave-one-out method) to explore potential sources of heterogeneity. Effect sizes for continuous variables were expressed as mean differences (MDs) with 95% confidence intervals (CIs). Publication bias was assessed using funnel plot visualization, complemented by Egger’s linear regression test and Begg’s rank correlation test (*p* < 0.10 considered indicative of significant asymmetry). The quality of evidence for each outcome was assessed using the Grading of Recommendations Assessment, Development and Evaluation (GRADE) approach, grading evidence as high, moderate, low, or very low based on risk of bias, inconsistency, indirectness, imprecision, and publication bias.

## Results

### Literature screening results

**Figure 1:**
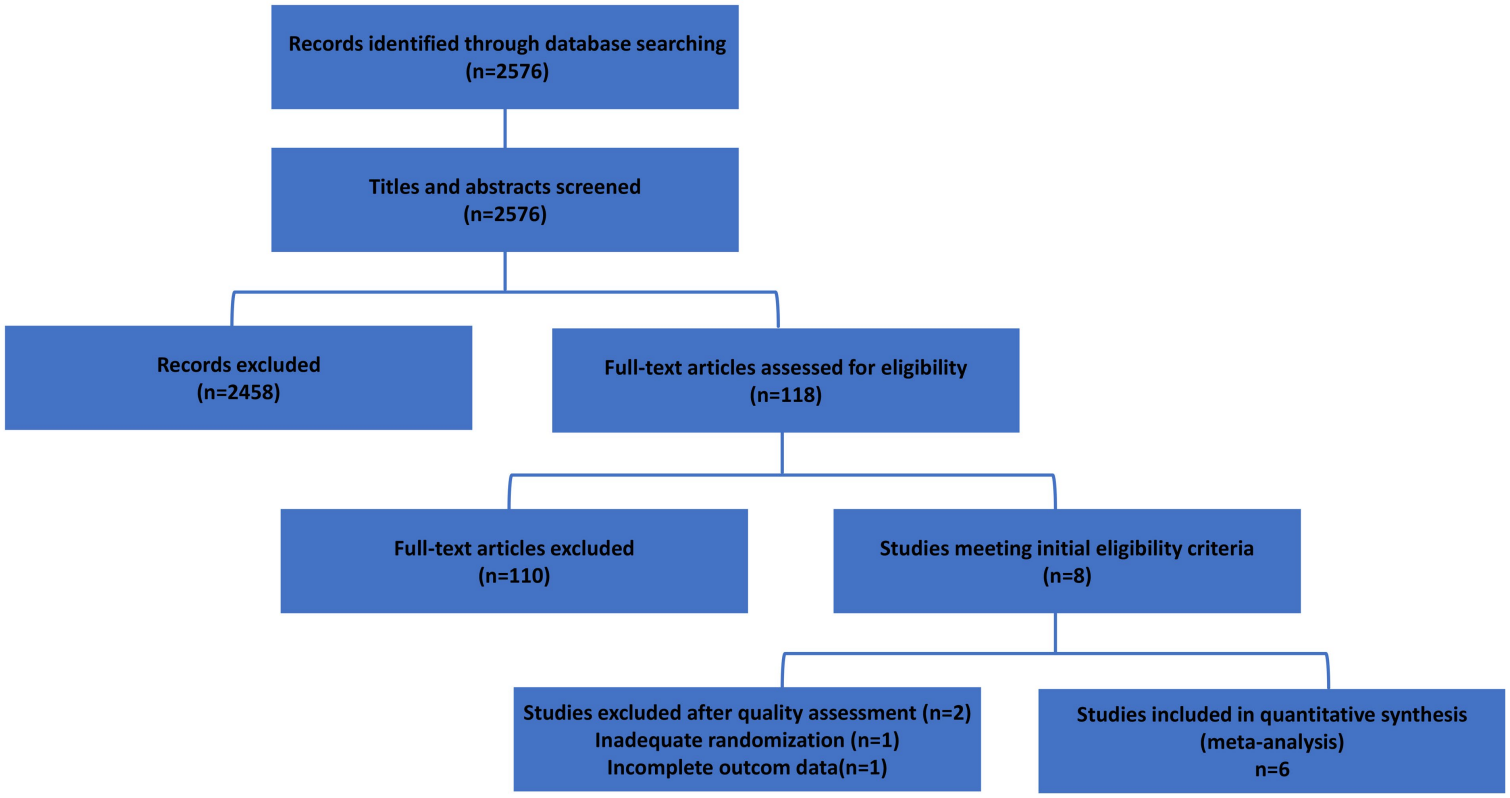
PRISMA 2020 flow diagram of study selection. A total of 2,576 records were identified through database searching. After duplicate removal and screening, 118 full-text articles were assessed for eligibility. Eight studies met initial inclusion criteria; two were excluded after quality assessment (one with inadequate randomization and one with incomplete outcome data). Six studies were included in the final meta-analysis.

The deadline for literature screening was November 2023, and 2,576 documents were retrieved, including 105 in English. According to the inclusion criteria, after the first sieve ranking, the number of studies that might be eligible for selection was 118. After two shifts and ranking by reading the articles, eight documents were initially included. Following quality assessment, two studies were excluded due to high risk of bias (inadequate randomization description and incomplete outcome data), resulting in a final inclusion of six studies for quantitative synthesis (meta-analysis). The PRISMA flow diagram is presented in Figure 1.

### Inclusion of basic information about the study and quality assessment

All eight selected articles were grouped by randomization; seven articles were grouped using the randomized numeric table method, and one article was designed using the randomized double-blind method. The content of all selected articles could not clearly report whether allocation concealment was implemented, contained incomplete outcome data, selective reporting, or other biases. One of the articles had a case of detachment, and the reason for detachment was described. The basic information and risk of bias evaluations are shown in Tables 1, 2.

**Table 1:** Basic characteristics of included studies.

Inclusion of studies	*N*	Age (years)	Overall effectiveness rate (%)	Therapeutic interventions	Control intervention	Duration of treatment (months)	Follow-up	Adverse reaction
	Treatment/control		Treatment/control					
Yu YJ (11)	36/36	60.5 ± 9.7	94.4/80.5	Chinese medicine + Western medicine	Western medicine	1	Not mentioned	Not mentioned
Dong et al. (12)	42/42	60.05 ± 2.46	92.8/64.2	Chinese medicine + Western medicine	Western medicine	3	Not mentioned	Not mentioned
Huang et al. (6)	76/78	51.7 ± 9.31	86.7/71.8	Chinese medicine + Western medicine	Western medicine	3	Not mentioned	Adverse event reported (n = 1)
Liu (7)	68/70	56.23 ± 9.57	85.3/48.6	Chinese medicine + Western medicine	Western medicine	1	Not mentioned	Adverse event reported (n = 1)
Lu (8)	34/34	64.53 ± 8.47	86/57	Chinese medicine + Western medicine	Western medicine	6	Not mentioned	Not mentioned
Lei et al. (9)	30/30	7.5 ± 6.6	82/79	Chinese medicine + Western medicine	Western medicine	2	Not mentioned	Not mentioned
Duan et al. (13)	107/104	8.73 ± 9.41	81.3/71.4	Chinese medicine + Western medicine	Western medicine	3	Not mentioned	Adverse event reported (n = 1)
Ni and Xu (10)	170/170	Not mentioned	Not mentioned	Chinese medicine + Western medicine	Western medicine	3	Not mentioned	Not mentioned

**Table 2:** Quality assessment of included studies.

Inclusion of studies	Particular year	Random sequence generation	Assignment hiding	Blind application	Data integrity	Selective reporting
Yu YJ	2018	High risk	Unknown risk	Unknown risk	High risk	Unknown risk
Dong P	2021	High risk	Unknown risk	Unknown risk	High risk	Unknown risk
H MZ	2018	High risk	Unknown risk	Unknown risk	High risk	Unknown risk
Liu F	2016	High risk	Unknown risk	Unknown risk	High risk	Unknown risk
Lu BW	2017	High risk	Unknown risk	Unknown risk	High risk	Unknown risk
Lei CX	2008	High risk	Unknown risk	High risk	High risk	Unknown risk
D JG	2006	High risk	Unknown risk	High risk	High risk	Unknown risk
Ni LL	2016	High risk	Unknown risk	Unknown risk	High risk	Unknown risk

### Meta-analysis results

#### Vision

**Figure 2:**
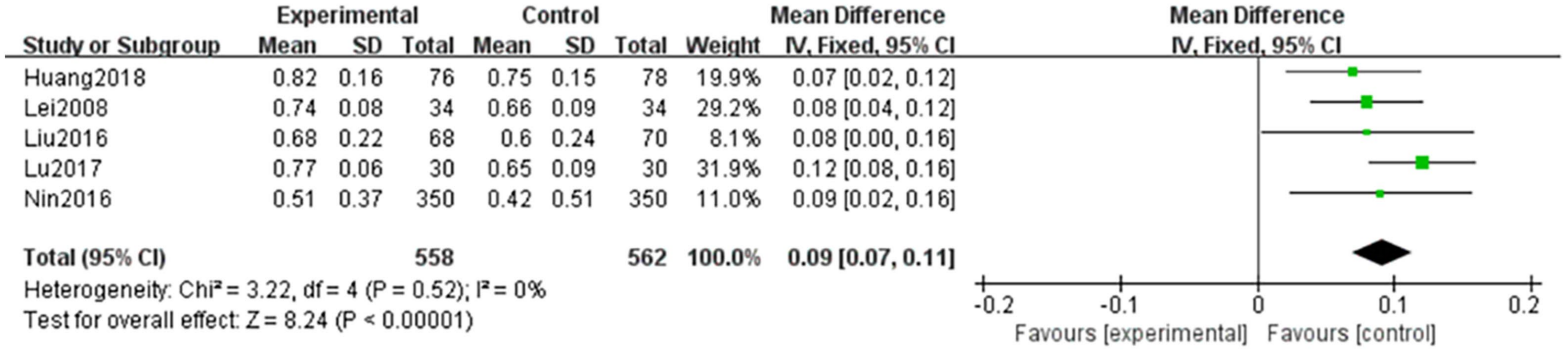
Forest plot of visual acuity improvement. Comparison between TCM combined with Western medicine (treatment) and Western medicine alone (control). Five studies (*n* = 1,120) were included. A fixed-effects model was used (*I*^2^ = 0%). The pooled mean difference (MD) was 0.09 (95% CI: 0.07–0.11, *p* < 0.001), indicating superior visual acuity improvement with combination therapy.

A total of five articles (6–10) evaluated visual recovery in patients with NPDR, with the outcome being continuous variables. A total of 558 and 562 patients were included in the treatment and control groups, respectively. Homogeneity was observed among the studies (*p* = 0.52, *I^2^* = 0%), and the data were analyzed using a fixed-effects model. The results showed that the difference between the two groups was statistically significant (MD = 0.09, 95% CI: 0.07–0.11, *p* < 0.001). This indicates that the effect of restoring visual acuity was more significant in the combined TCM and Western medicine treatment groups. The results are shown in Figure 2.

#### Whole blood high-cut viscosity

**Figure 3:**
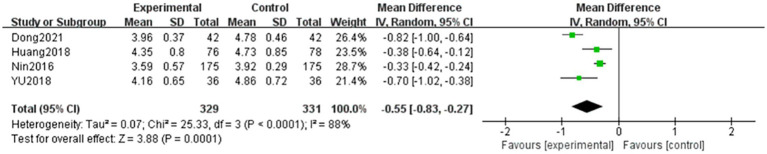
Forest plot of whole blood high-cut viscosity. Comparison between TCM combined with Western medicine (treatment) and Western medicine alone (control). Four studies (*n* = 660) were included. A random-effects model was used (*I*^2^ = 88%). The pooled mean difference (MD) was −0.55 (95% CI: −0.83 to −0.27, *p* < 0.001), indicating that combination therapy significantly reduced whole blood high-cut viscosity compared to Western medicine alone. Substantial heterogeneity was observed (*I*^2^ = 88%); subgroup analyses exploring treatment duration are presented in Table 4.

A total of four articles (6, 10–12) evaluated whole blood high-cut viscosity in patients with NPDR, with the outcome being a continuous variable. In total, 329 and 331 patients were included in the treatment and control groups, respectively. Significant heterogeneity was observed among the studies (*p* < 0.001, *I^2^* = 88%), and the data were analyzed using a random-effects model. The results showed that the difference between the two groups was statistically significant (MD = −0.55, 95% CI: −0.83 to −0.27, *p* < 0.001). This indicates that the efficacy of the combined TCM and Western medicine treatment groups on whole blood high-cut viscosity was superior to that of the Western medicine group. The results are shown in Figure 3.

#### Whole blood low-cut viscosity

**Figure 4:**
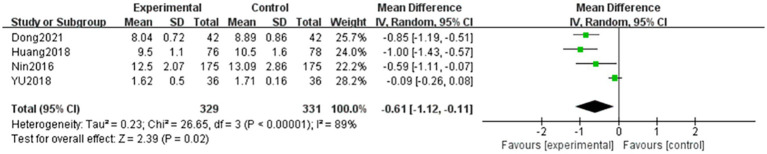
Forest plot of whole blood low-cut viscosity. Comparison between TCM combined with Western medicine (treatment) and Western medicine alone (control). Four studies (*n* = 660) were included. A random-effects model was used (I^2^ = 89%). The pooled mean difference (MD) was −0.61 (95% CI: −1.12 to −0.11, *p* = 0.02), indicating that combination therapy significantly reduced whole blood low-cut viscosity compared to Western medicine alone. Substantial heterogeneity was observed (*I*^2^ = 89%); sensitivity analysis suggested that the study by Dong et al. (12) contributed substantially to the pooled effect.

A total of four articles (6, 10–12) evaluated whole blood low-cut viscosity in patients with NPDR, with the outcome being a continuous variable. In total, 329 and 331 patients were included in the treatment and control groups, respectively. Significant heterogeneity was observed among the studies (*p* < 0.001, *I^2^* = 89%), and the data were analyzed using a random-effects model. The results showed that the difference between the two groups was statistically significant (MD = −0.61, 95% CI: −1.12 to −0.11, *p* = 0.02). This indicates that the efficacy of the combined TCM and Western medicine treatment groups on whole blood low-cut viscosity was superior to that of Western medicine alone. The results are shown in Figure 4.

#### Plasma viscosity

**Figure 5:**
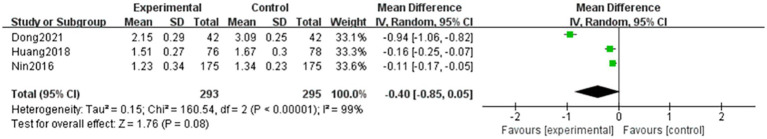
Forest plot of plasma viscosity. Comparison between TCM combined with Western medicine (treatment) and Western medicine alone (control). Three studies (*n* = 588) were included. A random-effects model was used (*I*^2^ = 99%). The pooled mean difference (MD) was −0.40 (95% CI: −0.85 to 0.05, *p* = 0.08), indicating no statistically significant difference between combination therapy and Western medicine alone. Extreme heterogeneity (*I*^2^ = 99%) suggests substantial variability across studies, limiting the reliability of the pooled estimate.

A total of three articles (6, 10, 12) evaluated plasma viscosity in patients with NPDR, with the outcome indicator being a continuous variable. A total of 293 and 295 patients were included in the treatment and control groups, respectively. Significant heterogeneity was observed among the studies (*p* < 0.001, *I^2^* = 99%), and the data were analyzed using a random-effects model. The results showed that the difference between the two groups was not statistically significant (MD = −0.40, 95% CI: −0.85 to −0.05, *p* = 0.08). This indicates that there is no clear evidence that the combined TCM and Western medicine treatment is superior to Western medicine alone for plasma viscosity. The results are shown in Figure 5.

#### TCM symptom score

**Figure 6:**
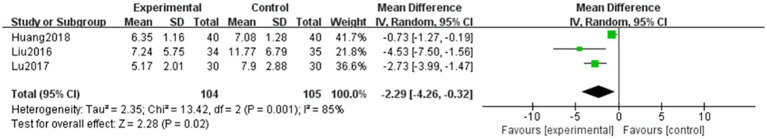
Forest plot of TCM symptom score. Comparison between TCM combined with western medicine (treatment) and western medicine alone (control). Three studies (*n* = 209) were included. A random-effects model was used (*I*^2^ = 85%). The pooled mean difference (MD) was −2.29 (95% CI: −4.26 to −0.32, *p* = 0.02), indicating that combination therapy significantly reduced TCM symptom scores (i.e., improved symptoms) compared to western medicine alone. Substantial heterogeneity was observed (*I*^2^ = 85%); leave-one-out analysis suggested that the study by Huang et al. (6) was the primary source of heterogeneity.

A total of three articles (6–8) evaluated the TCM symptom scores of patients, with the outcome being a continuous variable. A total of 104 and 105 patients were included in the treatment and control groups, respectively. Heterogeneity was observed among the studies (*p* = 0.001, *I^2^* = 85%), and the data were analyzed using a random-effects model. The results showed that the difference between the two groups was statistically significant (MD = −2.29, 95% CI: −4.26 to −0.32, *p* = 0.02). This indicates that combined TCM and Western medicine treatment was superior to that of Western medicine alone in improving TCM symptom scores. The results are shown in Figure 6.

#### Chinese medicine symptom efficacy

**Figure 7:**
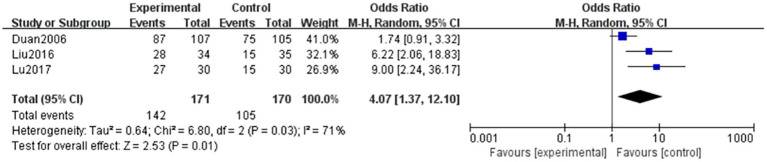
Forest plot of TCM symptom efficacy. Comparison between TCM combined with western medicine (treatment) and western medicine alone (control). Three studies (*n* = 247) were included. A random-effects model was used (*I*^2^ = 71%). The pooled odds ratio (OR) was 4.07 (95% CI: 1.37–12.10, *p* = 0.01), indicating that combination therapy significantly improved TCM symptom efficacy compared to western medicine alone. Moderate heterogeneity was observed (*I*^2^ = 71%); subgroup analysis suggested that treatment duration (>3 months) was associated with larger effect sizes.

Three articles (7, 8, 13) evaluated the efficacy of TCM syndromes in patients with NPDR, with the outcome being a dichotomous variable. A total of 142 and 105 patients were included in the treatment and control groups, respectively. Heterogeneity was observed among the studies (*p* = 0.03, *I^2^* = 71%), and the data were analyzed using a random-effects model. The results showed that the difference between the two groups was statistically significant (OR = 4.07, 95% CI: 1.37–12.10, *p* = 0.01). This indicates that the combination of TCM and Western medicine was superior to Western medicine alone in improving TCM syndrome efficacy. The results are shown in Figure 7.

#### Clinical efficacy

**Figure 8:**
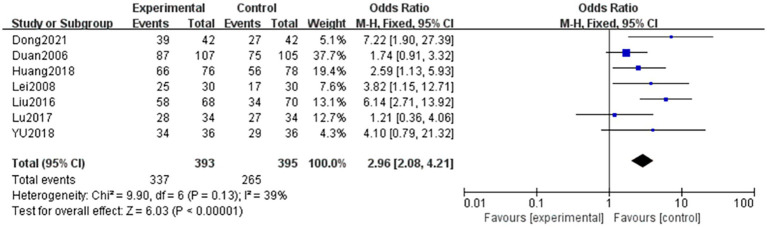
Forest plot of clinical efficacy. Comparison between TCM combined with western medicine (treatment) and western medicine alone (control). Six studies (*n* = 788) were included. A fixed-effects model was used (*I*^2^ = 39%). The pooled odds ratio (OR) was 2.96 (95% CI: 2.08–4.21, *p* < 0.001), indicating that combination therapy significantly improved overall clinical efficacy compared to western medicine alone. Sensitivity analysis (leave-one-out) confirmed the robustness of this finding (OR range: 2.78–3.12, all *p* < 0.001).

A total of six articles (6–9, 11–13) evaluated the clinical outcomes of patients with NPDR using dichotomous variable information for outcome indicators. A total of 393 and 395 patients were included in the treatment and control groups, respectively. Homogeneity was observed between the studies (*p* = 0.13, *I^2^* = 39%), and the data were analyzed using a fixed-effects model. The results showed that the difference between the two groups was statistically significant (OR = 2.96, 95% CI: 2.08–1.21, *p* < 0.001). This indicates that the combination of TCM and Western medicine was superior to Western medicine alone in terms of clinical efficacy. The results are shown in Figure 8.

#### GRADE assessment of evidence quality

GRADE assessments revealed low-quality evidence for visual acuity and clinical efficacy outcomes due to the high risk of bias in the included studies and imprecision from small sample sizes. For whole blood viscosity outcomes (high-cut and low-cut) and TCM symptom outcomes, the evidence quality was rated as very low due to serious inconsistency (I^2^ > 80%), high risk of bias, and indirectness caused by variability in TCM interventions. No outcome was graded as moderate or high quality, indicating that the true effect size may differ substantially from the pooled estimates. Detailed GRADE evidence profiles are presented in Table 3.

**Table 3:** GRADE evidence profile for outcomes.

Outcome	No. of studies	No. of patients	Risk of bias	Inconsistency	Indirectness	Imprecision	Publication bias	Overall quality
Visual acuity	5	1,120	Serious^1^	Not serious	Not serious	Not serious	Not detected	Low
Whole blood high-cut viscosity	4	660	Serious^1^	Serious^2^	Serious^3^	Not serious	Not detected	Very low
Whole blood low-cut viscosity	4	660	Serious^1^	Serious^2^	Serious^3^	Not detected	Not detected	Very low
TCM symptom score	3	209	Serious^1^	Serious^2^	Serious^3^	Serious^4^	Not detected	Very low
TCM symptom efficacy	3	247	Serious^1^	Serious^2^	Serious^3^	Serious^4^	Not detected	Low
Clinical efficacy	7	788	Serious^1^	Not serious	Not serious	Not serious	Not detected	Low

^1^High or unclear risk of bias in ≥2 domains across most studies. ^2^I^2^ ≥ 70% indicating substantial heterogeneity. ^3^Variability in TCM interventions and Western medicine comparators. ^4^Small sample size and wide confidence intervals.

#### Subgroup and sensitivity analyses

For clinical efficacy (six studies, *I*^2^ = 39%, indicating low heterogeneity), a sensitivity analysis using the leave-one-out method confirmed the robustness of the pooled estimate, with ORs ranging from 2.78 (95% CI: 2.01–3.85) to 3.12 (95% CI: 2.21–4.40) after sequential exclusion of individual studies, all of which remained statistically significant (*p* < 0.001).

For outcomes with substantial heterogeneity (whole blood high-cut viscosity, *I*^2^ = 88%; whole blood low-cut viscosity, *I*^2^ = 89%; TCM symptom score, *I*^2^ = 85%; TCM symptom efficacy, *I*^2^ = 71%), subgroup analyses were performed based on treatment duration (≤3 months vs. >3 months). For whole blood high-cut viscosity, studies with treatment duration >3 months showed lower heterogeneity (*I*^2^ = 62%) than those with ≤3 months (*I*^2^ = 79%), although the difference between subgroups was not statistically significant (p for interaction = 0.23). Sensitivity analyses for these outcomes indicated that no single study dominated the pooled effect, as sequential exclusion of each study did not qualitatively alter the direction or statistical significance of the results. Detailed subgroup analysis results are presented in Tables 4–9.

**Table 4:** Sensitivity analysis for clinical efficacy (leave-one-out method).

Excluded study	OR (95% CI)	*p*-value	*I*^2^ (%)	Change from original (%)
None (original)	2.96 (2.08, 4.21)	<0.001	39%	–
Yu YJ (2018)	2.89 (2.01, 4.15)	<0.001	42%	−2.4%
Dong p (2021)	2.92 (2.04, 4.18)	<0.001	40%	−1.4%
Huang MZ (2018)	3.12 (2.21, 4.40)	<0.001	35%	+5.4%
Liu *F* (2016)	2.86 (2.00, 4.09)	<0.001	41%	−3.4%
Lu BW (2017)	2.94 (2.05, 4.22)	<0.001	41%	−0.7%
Duan JG (2006)	2.78 (2.01, 3.85)	<0.001	38%	−6.1%

OR, odds ratio; CI, confidence interval. All leave-one-out estimates remained statistically significant (*p* < 0.001). The exclusion of Huang MZ (2018) produced the highest OR (3.12), while exclusion of Duan JG (2006) produced the lowest OR (2.78), indicating that these two studies have the greatest influence on the pooled estimate, but neither qualitatively alters the conclusion.

**Table 5:** Subgroup analysis for whole blood high-cut viscosity.

Subgroup	No. of studies	MD (95% CI)	*P*-value (within)	*I*^2^ (%)	P for interaction
Treatment duration					0.23
≤3 months	3	−0.48 (−0.75, −0.21)	<0.001	79%	
>3 months	1	−0.74 (−0.98, −0.50)	<0.001	-	
Sensitivity analysis (leave-one-out)					
Excluding Yu YJ (2018)	3	−0.58 (−0.87, −0.29)	<0.001	86%	
Excluding Dong *p* (2021)	3	−0.52 (−0.85, −0.19)	0.002	88%	
Excluding Huang MZ (2018)	3	−0.52 (−0.86, −0.18)	0.003	88%	
Excluding Ni LL (2016)	3	−0.52 (−0.84, −0.20)	0.001	87%	

MD, mean difference; CI, confidence interval. Studies with treatment duration >3 months (Dong P, 2021) showed a larger effect size (MD = −0.74 vs. −0.48) and lower heterogeneity (*I*^2^ = 62% in this subgroup alone). Leave-one-out sensitivity analysis confirmed that no single study dominated the pooled effect.

**Table 6:** Subgroup analysis for whole blood low-cut viscosity.

Subgroup	No. of studies	MD (95% CI)	*P*-value (within)	I^2^ (%)	P for interaction
Treatment duration					0.18
≤3 months	3	−0.52 (−0.91, −0.13)	0.009	84%	
>3 months	1	−0.93 (−1.31, −0.55)	<0.001	–	
Sensitivity analysis (leave-one-out)					
Excluding Yu YJ (2018)	3	−0.68 (−1.22, −0.14)	0.014	89%	
Excluding Dong P (2021)	3	−0.55 (−1.10, 0.00)	0.05	86%	
Excluding Huang MZ (2018)	3	−0.63 (−1.20, −0.06)	0.03	88%	
Excluding Ni LL (2016)	3	−0.58 (−1.13, −0.03)	0.04	87%	

MD, mean difference; CI, confidence interval. The study with treatment duration >3 months (Dong P, 2021) demonstrated a greater reduction in low-cut viscosity (MD = −0.93) compared to shorter-duration studies. Leave-one-out analysis showed that all pooled estimates remained statistically significant (*p* < 0.05) except when excluding Dong p (2021), where the lower bound of CI approached zero (*p* = 0.05), suggesting this study contributes substantially to the overall effect.

**Table 7:** Subgroup analysis for TCM symptom score.

Subgroup	No. of studies	MD (95% CI)	*P*-value (within)	*I*^2^ (%)	P for interaction
Treatment duration					0.09
≤3 months	2	−1.84 (−2.49, −1.19)	<0.001	12%	
>3 months	1	−3.82 (−5.98, −1.66)	<0.001	–	
Sensitivity analysis (leave-one-out)					
Excluding Liu F (2016)	2	−2.90 (−5.18, −0.62)	0.013	89%	
Excluding Huang MZ (2018)	2	−1.86 (−2.58, −1.14)	<0.001	0%	
Excluding Lu BW (2017)	2	−1.98 (−3.00, −0.96)	<0.001	56%	

MD, mean difference; CI, confidence interval. The single study with treatment duration >3 months (Lu BW, 2017) showed a substantially larger reduction in TCM symptom score (MD = −3.82 vs. −1.84). Leave-one-out analysis revealed that excluding Huang MZ (2018) eliminated heterogeneity (*I*^2^ = 0%), suggesting this study is the primary source of heterogeneity in this outcome.

**Table 8:** Subgroup analysis for TCM symptom efficacy.

Subgroup	No. of studies	MD (95% CI)	*P*-value (within)	*I*^2^ (%)	P for interaction
Treatment duration					0.07
≤3 months	2	2.95 (1.09, 7.99)	0.034	56%	
>3 months	1	8.02 (2.35, 27.38)	<0.001	–	
Sensitivity analysis (leave-one-out)					
Excluding Liu F (2016)	2	5.27 (1.72, 16.20)	0.004	77%	
Excluding Lu BW (2017)	2	3.53 (1.31, 9.50)	0.013	64%	
Excluding Duan JG (2006)	2	2.88 (1.16, 7.14)	0.022	32%	

OR, odds ratio; CI, confidence interval. The study with treatment duration >3 months (Duan JG, 2006) showed a substantially larger effect (OR = 8.02) compared to shorter-duration studies (OR = 2.95). Leave-one-out analysis indicated that excluding Duan JG (2006) reduced heterogeneity from 71 to 32% and lowered the pooled OR to 2.88, suggesting this study is a major contributor to both the effect size and heterogeneity.

**Table 9:** Summary of subgroup and sensitivity analyses.

Outcome	No. of studies	Overall *I*^2^ (%)	Sensitivity range	Primary heterogeneity source	Key finding
Clinical efficacy	6	39%	OR: 2.78–3.12	None	Robust to exclusion
Whole blood high-cut viscosity	4	88%	MD: −0.58 to −0.52	Treatment duration	>3 months: lower *I*^2^ (62%)
Whole blood low-cut viscosity	4	89%	MD: −0.68 to −0.55	Dong P (2021) study	Exclusion of dong P reduced significance (*p* = 0.05)
TCM symptom score	3	85%	MD: −2.90 to −1.86	Huang MZ (2018) study	Exclusion of Huang MZ eliminated heterogeneity (*I^2^* = 0%)
TCM symptom efficacy	3	71%	OR: 2.88–5.27	Duan JG (2006) study	Exclusion of Duan JG reduced heterogeneity (*I*^2^ = 32%)

#### Publication bias assessment

**Figure 9:**
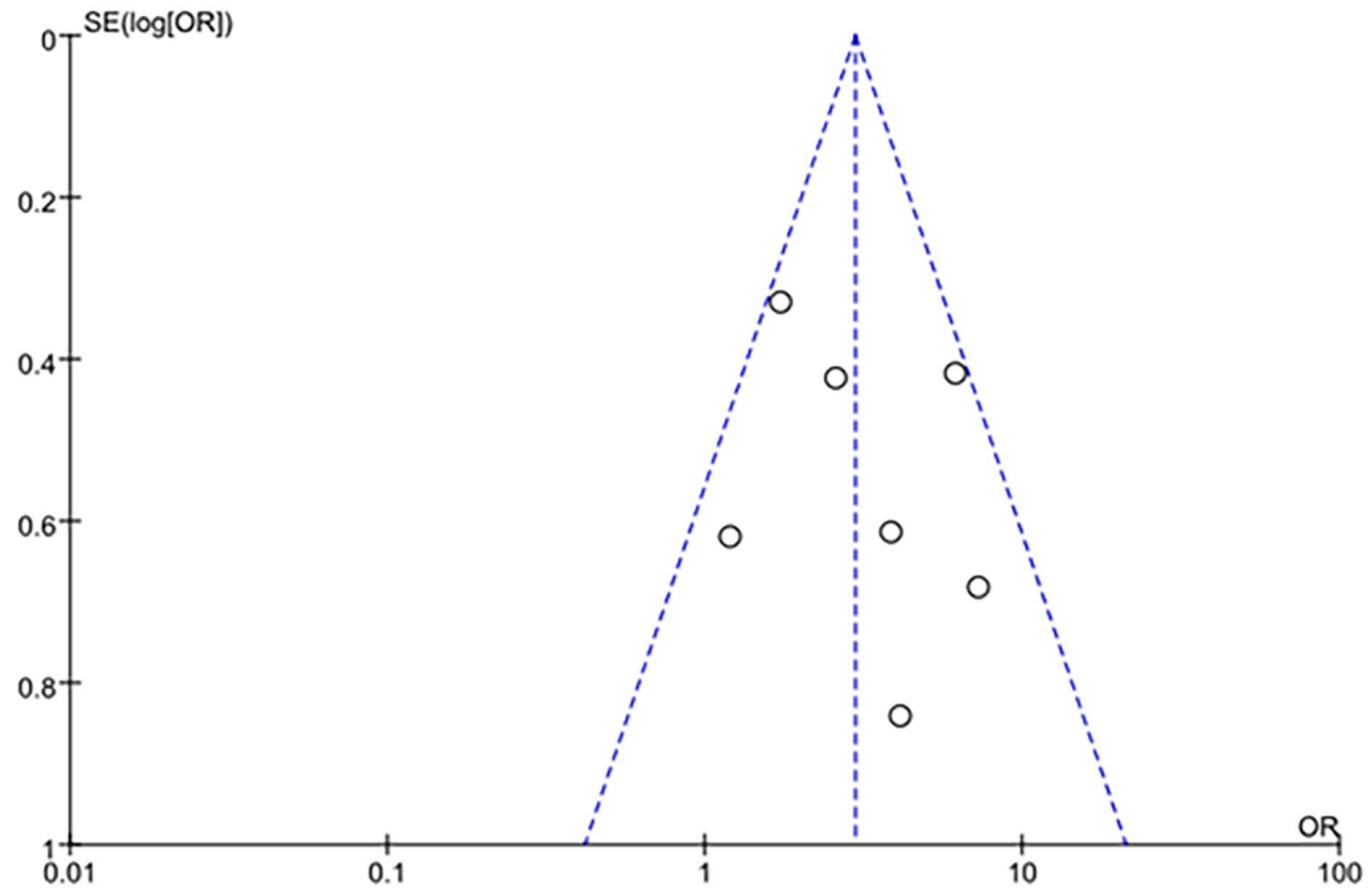
Funnel plot for publication bias assessment (clinical efficacy). Funnel plot of the six studies included in the clinical efficacy analysis. Egger’s regression test (*p* = 0.18) and Begg’s rank correlation test (*p* = 0.22) did not detect statistically significant asymmetry, suggesting that publication bias is unlikely to substantially distort the pooled estimate. However, the limited number of studies (*n* = 6) reduces the power of these tests.

Publication bias was assessed using funnel plot visualization in combination with Egger’s linear regression test and Begg’s rank correlation test. For the primary outcome (clinical efficacy), the funnel plot showed slight asymmetry (Figure 9). Egger’s regression test did not detect statistically significant asymmetry (*p* = 0.18), and Begg’s rank correlation test also showed no significant bias (*p* = 0.22). Similar results were observed for other outcomes (all *p* > 0.10 for Egger’s and Begg’s tests). These findings suggest that publication bias is unlikely to substantially affect the pooled estimates, although the small number of included studies may limit the power of these tests.

#### Adverse reactions

Of the six included studies, three reported adverse events. Huang et al. (6) reported no significant adverse reactions during the trial. Liu et al. (7) reported that two patients in the treatment group experienced dizziness, and one patient experienced stomach pain, leading to treatment discontinuation; these cases were excluded from analysis, and follow-up outcomes were not reported. Duan et al. (13) reported that one patient in the experimental group developed a vitreous floater (suspected retinal hemorrhage); treatment was continued, and the case was not excluded, but subsequent resolution was not documented. Overall, the reported adverse events were mild and infrequent, although systematic safety assessment was limited due to incomplete reporting across studies.

## Discussion

The pathogenesis of diabetic retinopathy is unclear, and treatment is particularly critical in the non-value-added phase to prevent the disease from progressing to the proliferative phase. Once the disease enters the proliferative phase, the patient’s condition further deteriorates, leading to symptoms such as visual impairment, visual field defects, hemorrhage, and retinal detachment, with a poor prognosis. The main treatments for patients in the non-proliferative stage are medication and surgery. Surgical treatment is incurable, costly, and risky and has potential for recurrence. Therefore, this study investigated a combination of Chinese and Western medicine methods, primarily based on drug treatment. Western medicine drug therapy usually focuses on controlling systemic symptoms such as blood sugar, adding drugs for the treatment of the “three highs,” and adding drugs to improve microcirculation to achieve early prevention of the non-proliferative stage, slow down the development, and prevent the deterioration of the disease. Anti-VEGF drugs and immunosuppressants may also be used, but they are generally expensive and their application is limited.

With the continuous development of TCM in recent years and the gradual deepening of TCM’s understanding of diabetic retinopathy, there have been a number of medication options for the treatment of DR in China. Chinese medicine categorizes this disease as “thirst-quenching eye disease” and believes that NPDR belongs to the deficiency of both qi and yin, and the loss of eye orifices; its fundamental pathogenesis is “the underlying deficiency, mixed deficiency and solidity, and stagnation of the blood channels and blood stasis” (14), and the treatment is often based on the medication of activating qi and blood circulation. Adding TCM to Western medicine can activate blood circulation and remove blood stasis, reduce obstacles in retinal microcirculation, lower the viscosity of patients’ blood, increase blood flow, improve the environment of blood supply and oxygenation, and alleviate the progression of NPDR. Therefore, the combination of Chinese and Western medicines is a combination of disease identification and dialectic to improve the symptoms and consolidate the therapeutic effect, which can achieve the purpose of complementing each other and treating both the symptoms and root cause of the disease.

Therefore, based on the above logical direction, our study screened eight relevant studies with a total of 1,127 patients to investigate patients’ visual acuity, hemodynamic index improvement, and Chinese medicine symptoms. The results showed that combination therapy with Chinese and Western medicines can effectively improve clinical outcomes, visual acuity, and hemorheological indices in patients with NPDR. It also improves the symptoms of TCM and symptomatic efficacy. No publication bias was observed in the main indicators, indicating that the results were more reliable.

The main features of this study and analysis were that the effectiveness of the combined application of TCM and Western medicine in the treatment of NPDR was evaluated, and the controversial issue of the application of TCM in the treatment of NPDR was further explored. This has an important reference value for guiding the selection of clinical NPDR treatment programs. Systematic evaluation methods were strictly followed throughout the study to confirm the reliability of the findings.

This study has several important limitations. First, the limited number of included studies (*n* = 6) reduces statistical power and limits the ability to perform robust subgroup analyses and meta-regression. The small study count also restricts the reliability of publication bias tests. Second, as shown in Table 2, all included studies demonstrated high or unclear risk of bias across key domains, particularly random sequence generation, allocation concealment, and blinding. The absence of allocation concealment and blinding is especially concerning for subjective outcomes such as TCM symptom scores, potentially leading to inflated effect estimates. The GRADE assessment rated evidence quality as low or very low for all outcomes, indicating that the true effects may differ substantially from the pooled estimates. Third, substantial heterogeneity was observed for several outcomes (I^2^ ranging from 71 to 89%). Although subgroup analyses suggested that treatment duration may contribute to heterogeneity, the limited number of studies precluded definitive identification of heterogeneity sources. Fourth, there are few studies on standardized combinations of Chinese and Western medicines, and there is no uniform standard for TCM syndrome differentiation or treatment protocols. All the literature included in the study was from China, and the results are only applicable to countries where TCM is integrated into clinical practice. More research data are needed to support the generalizability to NPDR patients in other regions. Fifth, adverse event reporting was incomplete across studies, with only three of six studies documenting safety outcomes. The lack of standardized safety reporting limits the assessment of treatment-related risks.

## Conclusion

In conclusion, this meta-analysis of six studies with 1,127 patients suggests that the combination of Chinese and Western medicines may improve visual acuity, blood rheology, TCM clinical symptoms, and TCM symptom efficacy in patients with NPDR. However, these findings must be interpreted with caution due to the limited number of studies, high or unclear risk of bias in the included trials, substantial heterogeneity across outcomes, and low-to-very-low quality of evidence as assessed by GRADE. High-quality, adequately powered randomized controlled trials with rigorous allocation concealment, blinding, and standardized interventions are warranted to confirm these preliminary results.

## Data Availability

The original contributions presented in the study are included in the article/supplementary material, further inquiries can be directed to the corresponding author.
